# Machine learning-based fusion model for predicting HER2 expression in breast cancer by Sonazoid-enhanced ultrasound: a multicenter study

**DOI:** 10.3389/fmed.2025.1585823

**Published:** 2025-05-21

**Authors:** Huiting Zhang, Manlin Lang, Huiming Shen, Hang Li, Ning Yang, Bo Chen, Yixu Chen, Hong Ding, Weiping Yang, Xiaohui Ji, Ping Zhou, Ligang Cui, Jiandong Wang, Wentong Xu, Xiuqin Ye, Zhixing Liu, Yu Yang, Tianci Wei, Hui Wang, Yuanyuan Yan, Changjun Wu, Yiyun Wu, Jingwen Shi, Yaxi Wang, Xiuxia Fang, Ran Li, Ping Liang, Jie Yu

**Affiliations:** ^1^Department of Interventional Ultrasound, PLA Medical College & Chinese PLA General Hospital, Beijing, China; ^2^Department of Ultrasound, Zhongda Hospital, Nanjing, China; ^3^Department of Breast Surgery, Affiliated Hospital of Putian University, Putian, China; ^4^Department of Ultrasound, Xingcheng People’s Hospital, Xingcheng, China; ^5^Department of Ultrasound Medicine, Lu’ an People’s Hospital of Anhui Province, Liuan, China; ^6^Department of Ultrasound, The Fifth People's Hospital of Chengdu, Chengdu, China; ^7^Department of Ultrasound, Huashan Hospital, Shanghai, China; ^8^Department of Ultrasound, Guangxi Medical University Cancer Hospital, Nanning, China; ^9^Department of Ultrasound, The Fourth Hospital of Hebei Medical University, Shijiazhuang, China; ^10^Department of Ultrasound, The Third Xiangya Hospital, Changsha, China; ^11^Department of Ultrasound, Peking University Third Hospital, Beijing, China; ^12^General Surgery, Chinese PLA General Hospital, Beijing, China; ^13^Department of Ultrasound, First Affiliated Hospital of Southern University of Science and Technology, Second Clinical College of Jinan University, Shenzhen Medical Ultrasound Engineering Center, Shenzhen People’s Hospital, Shenzhen, China; ^14^Department of Ultrasound Medicine, The First Affiliated Hospital of Nanchang University, Nanchang, China; ^15^Department of Ultrasound, Beijing Friendship Hospital, Beijing, China; ^16^Department of Ultrasound, The 2nd Affiliated Hospital of Harbin, Harbin, China; ^17^Department of Ultrasound, China-Japan Union Hospital of Jilin University, Changchun, China; ^18^Department of Ultrasound, Zhengzhou Central Hospital, Zhengzhou, China; ^19^Department of Ultrasound, The First Affiliated Hospital of Harbin Medical University, Harbin, China; ^20^Department of Ultrasound, Affiliated Hospital of Nanjing University of Chinese Medicine, Nanjing, China; ^21^Department of Ultrasound, Shengjing Hospital of China Medical University, Shenyang, China; ^22^Department of Ultrasound, The Affiliated Hospital of Inner Mongolia Medical University, Hohhot, China; ^23^Department of Ultrasound, The First Affiliated Hospital of Xinxiang Medical University, Xinjiang, China

**Keywords:** human epidermal growth factor receptor 2, breast cancer, Sonazoid, ultrasound, machine learning

## Abstract

**Purpose:**

To predict human epidermal growth factor receptor 2 (HER2) expression in breast cancer (BC) using Sonazoid-enhanced ultrasound in a machine learning-based model.

**Materials and methods:**

Between August 2020 and February 2021, patients with breast cancer who underwent surgical treatment without neoadjuvant chemotherapy were prospectively enrolled from 17 hospitals in China. HER2 expression status was assessed by immunohistochemistry or fluorescence *in situ* hybridization (FISH). The training set contained data from 11 hospitals and the validation set contained 6 hospitals. Clinical features, B-mode ultrasound, contrast-enhanced ultrasound (CEUS), and time-intensity curve were selected by the Least Absolute Shrinkage and Selection Operator. Based on the selected features, six prediction models were established to predict HER2 3 + and 2 +/1 + expression: logistic regression (LR), support vector machine (SVM), random forest (RF), eXtreme Gradient Boosting (XGB), XGB combined with LR, and fusion model.

**Results:**

A total of 140 patients with breast cancer were enrolled in this study. Seven features related to HER2 3 + and six features related to HER2 2+/1 + were selected to establish prediction models. Among the six models, LR, SVM, and XGB showed the best prediction performance for both HER2 3 + and HER2 2+/1 + cases. These three models were then combined into a fusion model. In the validation, the fusion model achieved the highest value of area under the receiver operating characteristic curve as 0.869 (95%CI: 0.715–0.958) for predicting HER2 3 + and 0.747 (95%CI: 0.548–0.891) for predicting HER2 2+/1 + cases. The model could correctly upgrade HER2 2 + cases to HER2 3 + cases, consistent with the FISH test results.

**Conclusion:**

Sonazoid-enhanced ultrasound can provide effective guidance for targeted therapy of breast cancer by predicting HER2 expression using machine learning approaches.

## 1 Introduction

According to the World Health Organization, breast cancer (BC) can cause 500,000 deaths, and 1.7 million new cases are diagnosed annually (1). Characterized by overexpression of the human epidermal growth factor receptor 2 (HER2) gene and its protein, HER2-positive breast cancer accounts for 20–30% of breast cancer cases and requires distinct therapeutic strategies (2, 3). Trastuzumab and pertuzumab, which are targeted by monoclonal antibody therapies, improve the survival outcomes of HER2-positive (HER2 3+) breast cancer (4–6). Recent reports have recommended HER2-targeted agents and antibody-drug conjugates (ADCs) as new clinical therapies for HER2-low expression (HER2 1+, 2+) breast cancer (7). The distinct pathological characteristics of HER2 0, HER2-low, and HER2-positive breast cancers have been the focus of research. Studies have reported that the 50% recurrence rate of HER2-positive breast cancers can be decreased by the use of HER2-targeted monoclonal antibodies (8).

For patients with HER2-positive cancers, preoperative targeted therapy could increase the chance of breast conservation and sentinel lymph node biopsy rather than mastectomy and axillary lymph node dissection (7, 9). The selection of breast cancer neoadjuvant treatment regimens (particularly monoclonal antibodies) depends on the results of preoperative core needle biopsy (CNB), especially molecular profiling tested by immunohistochemistry (IHC) and fluorescence *in situ* hybridization (FISH) (10–12). However, because of intratumoral heterogeneity, the inadequate tissue acquired from CNB may not provide complete pathological characteristics of the tumor, causing discordance between cores in 8% of HER2-positive cases and discordance between CNB and surgical pathology results for approximately 26.6% of HER2 status (11, 13, 14). Thus, HER2 expression levels in breast cancer could be underestimated, and the concomitant false-negative results may cause missed diagnosis of HER2-positive cases, affecting clinical arrangements and prognosis. Increasing the number of multi-point punctures may increase the accuracy or decrease the underestimation in the diagnosis of HER2 expression. However, it has been reported that the possibility of core needle seeding in breast cancer varies from 2 to 63% (15–17). Adding the number of punctures to increase the amount of tissue may also increase the risk of tumor seeding (16, 17).

Contrast-enhanced ultrasound (CEUS) indicates vascular information of the tumor, which has been widely used in the diagnosis of benign and malignant breast lesions, assessing the pathological characteristics, and predicting neoadjuvant chemotherapy (NAC) response (18, 19). CEUS can improve the categorization of suspicious breast lesions, reduce unnecessary biopsies, and improve the cancer yield rate of biopsy procedures (20). SonoVue (Bracco, Milan, Italy), the most widely used ultrasound contrast agent, consisting of sulfur hexafluoride microbubbles, has shown better performance in low-intensity imaging (21). Consisting of lipid-stabilized perfluorocarbon microbubbles, Sonazoid (GE Healthcare, Oslo, Norway) is more stable for long-term imaging and has a higher resistance to ultrasound mechanical index (MI), which is more suitable for high-frequency linear array probe scanning (22, 23). Machine learning approaches have been widely applied for the early detection, diagnosis, and outcome prediction of breast cancer (24, 25). It has been reported that the diagnostic accuracy and sensitivity of CEUS in breast cancer can be improved by combining it with a machine learning approach (20).

Hence, our study aims to predict the HER2 status of breast cancer by combining B-mode ultrasound and contrast Sonazoid-enhanced ultrasound features using machine learning models.

## 2 Materials and methods

### 2.1 Patients

This prospective, multicenter study was approved by the institutional ethics committee (ClinicalTrials.gov: NCT04657328). Informed written consent was obtained from all participants before the examinations. Between August 2020 and February 2021, 168 patients with breast cancer with 168 breast masses diagnosed by surgical pathology from a multicenter cohort of 17 hospitals in China, were enrolled in this study. Patients with an unclear HER2 status and incomplete time-intensity curve (TIC) features were excluded. According to current guidelines (26), HER2 status was determined using IHC for HER2 protein expression and FISH for equivocal cases (IHC 2+). The multicenter IHC results for HER2 expression were evaluated by experienced pathologists. A total of 140 patients with HER2 status were included in the study. The exclusion criteria were (1) absence of HER2 results and (2) absence of TIC features due to substandard image acquisition. The training set contained datasets from 11 hospitals, including 104 and 79 cases in the two cohorts. The external validation set contained prospective datasets from 6 other hospitals, including 36 and 28 cases in the two cohorts. Among these cases, 104 patients from 11 hospitals were included in the training set and 36 patients from the other 6 hospitals were included in the validation set. In total, there were 26 HER2-positive (IHC 3+), 68 HER2-low (39 IHC 1 + and 29 IHC 2+), 39 HER2 0 (IHC 0), and 7 HER2-negative (IHC 0, 1+, and 2+) cases in the training and validation sets. In total, 88 patients with invasive ductal carcinoma, 3 with mucinous breast carcinoma, 1 with metaplastic breast carcinoma, and 12 with ductal carcinoma *in situ* were included.

**Figure 1:**
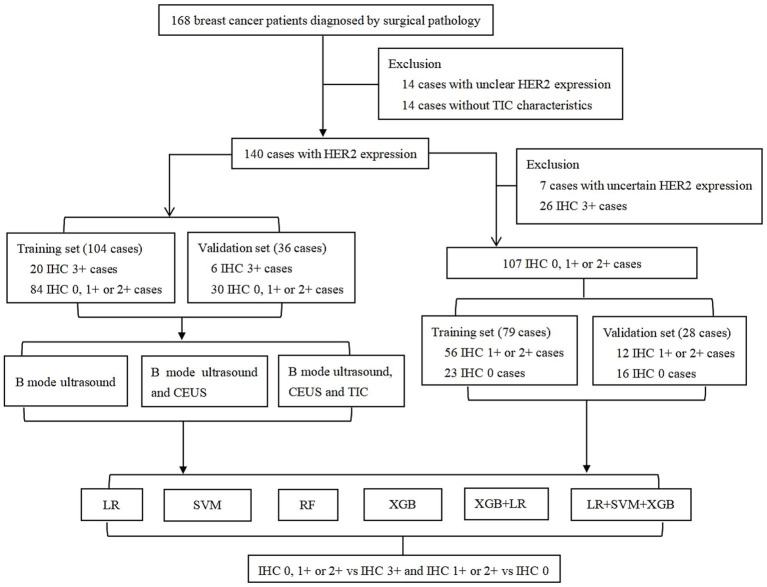
Flowchart of study design. HER2: human epidermal growth factor receptor-2; IHC: immunohistochemistry; TIC: time intensity curve; CEUS: contrast-enhanced ultrasound. LR: logistic regression; SVM: support vector machine; RF: random forest; XGB: XGBoost.

Furthermore, to differentiate HER2-low expression cases from HER2 0 and exclude the confounding effect of HER2-positive expression levels in the analysis, 26 HER2-positive cases and 7 patients with uncertain HER2 expression status (only known as HER2-negative cases) in the cohort were excluded. Finally, 107 patients were included in the HER2-negative and low-expression group, containing 79 patients in the training cohort from the same 11 hospitals and 28 patients in the validation cohort. The study design is shown in Figure 1.

### 2.2 B-mode and CEUS image acquisition

**Figure 2:**
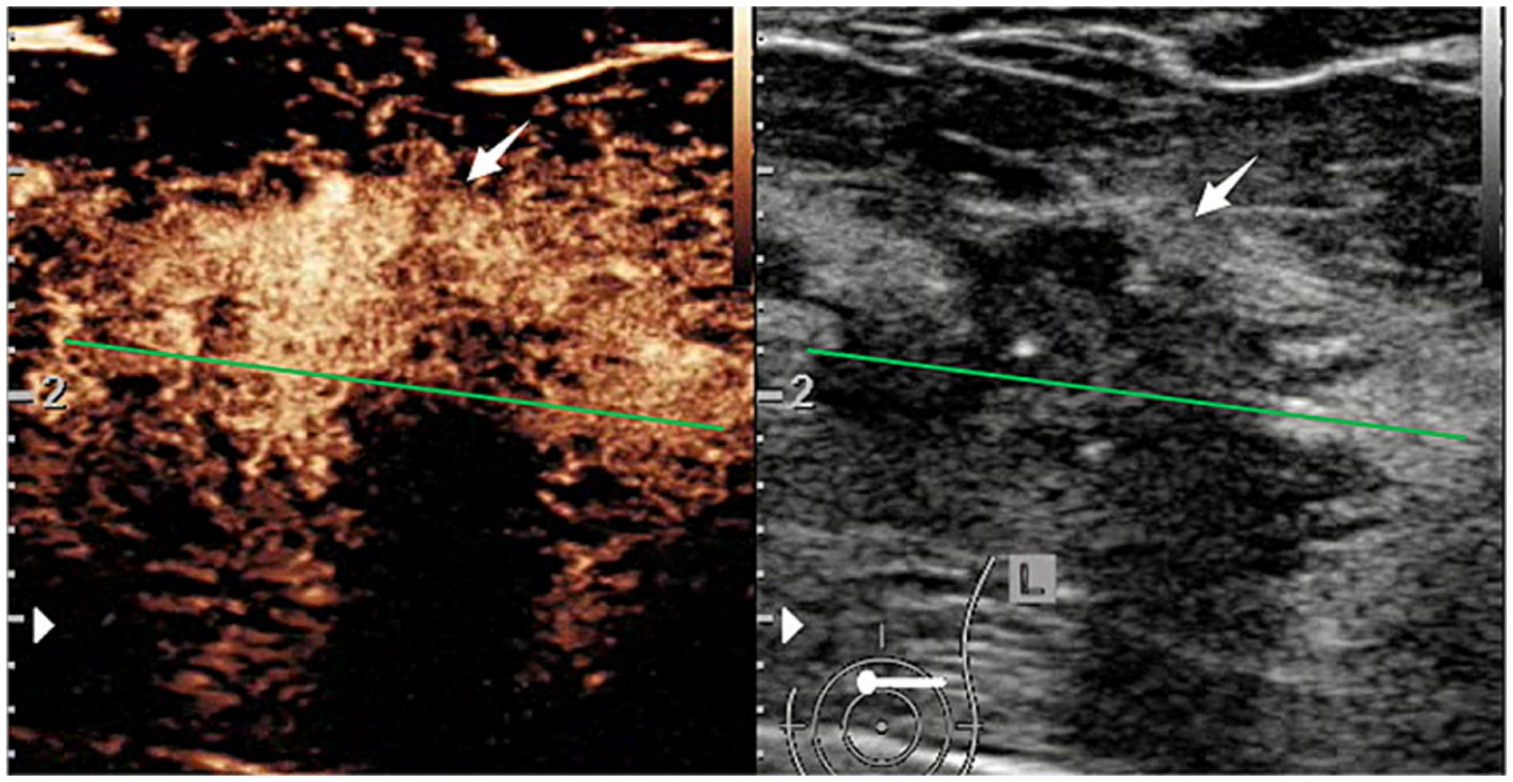
CEUS and B-mode ultrasound images of a patient with HER2-positive breast cancer.

B-mode ultrasound and CEUS examinations were performed by radiologists from 17 hospitals with 10 ultrasound devices (Supplementary Table 1) equipped with a linear probe. All ultrasound examinations were conducted following a uniform diagnostic consensus. Prior to image acquisition, participating radiologists in this multicenter study, with more than 3 years of experience in breast ultrasound, received systematic training in B-mode and CEUS breast examination. All radiologists in this study received standardized training in breast CEUS interpretation according to Sonazoid instructions and previous studies. They were required to complete a minimum of 50 breast CEUS-independent case evaluations to ensure consistent diagnostic consensus prior to the study. Breast masses were first identified using a B-mode ultrasound scan. Next, 0.015 mL/kg of perfluorobutane-filled microbubble contrast agent (Sonazoid; GE Healthcare, Oslo, Norway) was injected via the catheter line (≥ 22-gauge) placed in the antecubital vein, followed by a 5 mL flush of 0.9% sodium chloride solution. The mechanical index of 0.18–0.24 was applied. When the injection was completed, the imaging timer was started simultaneously. After 1 min of continuous assessment of the whole mass, intermittent scanning (10 s each time) was arranged at the time points of 1.5 min, 2 min, 3 min, 4 min, and 5 min. For patients with multiple masses, images of the largest masses were preserved. Both B-mode and CEUS images and videos were stored in DICOM format on a hard disk at the hospital and sent to our study center. Finally, six radiologists with more than 15 years of experience in conventional breast ultrasound and breast CEUS were independently evaluated for image features at the study center (Figure 2).

In B-mode breast ultrasound, the “strip-shaped echoic” feature represents thin, elongated, and hyperechoic lines or bands within the breast tissue or mass. CEUS characteristics were evaluated, including shape (regular or not), margin (well or poorly defined), wash-in time (earlier, later, synchronous), enhancement degree (hyperenhancement, isoenhancement, hypoenhancement), complete wash-out time of lesions (≤5 min or not), uptake pattern (centripetal, centrifugal, diffuse, no enhancement), as well as exhibitions of the homogeneous pattern, rim-like enhancement, claw-shaped pattern, perfusion defects, lesion size compared with conventional ultrasound increased, and nourishing vessels. The time-intensity curve (TIC) features were evaluated using external perfusion software (VueBox^™^) to quantitatively evaluate the microvasculature of the tumors through the CEUS videos.

### 2.3 Statistical analysis

R version 3.4.4 software, SPSS Version 23.0 (IBM, Armonk, NY, United States), and MedCalc 19.5.6 were used to perform statistical analysis. Statistics are described as mean ± standard deviation or numbers with percentiles for distribution. The *t*-test, chi-square test, and the Least Absolute Shrinkage and Selection Operator (LASSO) were used to select the features. The regularization property of LASSO constrains the model coefficients through the penalty parameter (*λ*) and shrinks the coefficients of less important variables to zero to mitigate overfitting (27, 28). Logistic regression (LR), support vector machine (SVM), random forest (RF), eXtreme Gradient Boosting (XGB), late fusion model based on the voting method, and XGB combined with LR were trained to classify HER2-positive status and HER2 low expression status in the two groups. A combination of XGB (constructing new features based on existing features) and LR (classifiers) was used to establish the prediction model. Prediction models were established on the training set, and their performance was tested on the validation set (29). For internal validation, leave-one-out cross-validation (LOOCV) was performed to assess the predictive accuracy and stability of the training set. External validation was performed to test the performance of the trained models, evaluate their generalizability, and identify potential biases. The receiver operating characteristic curves (ROC) of the predictive models were analyzed. The area under the receiver operating characteristic curve (AUC), accuracy, sensitivity, specificity, and 95%CI were assessed. The DeLong test was used to compare differences between the AUC values of the different models.

## 3 Results

### 3.1 Clinical characteristics

The clinical characteristics of 140 patients with breast cancer (mean age 52.35 ± 11.03 years, range 23–85 years) with 140 masses are shown in Table 1. In the training cohort, 104 patients were enrolled, including 20 HER2-positive cases. Of the 107 patients in the HER2 low expression group, 79 were included in the training cohort, with 56 IHC 2 + or 1 + and 23 IHC 0 cases.

**Table 1:** Clinical characteristics of 140 patients in training and validation sets.

	Total (*n* = 140)	Training set (*n* = 104) (%)	Validation set (*n* = 36) (%)	*p* value
Age (years)	52.35 ± 1.03 (23–85)	53.13 ± 11.07	50.11 ± 10.75	0.158
BMI (kg/m^2^)	24.20 ± 5.04 (13.65–63.70)	24.46 ± 5.30	23.46 ± 4.18	0.308
Menopause				0.608
Premenopause	61	44 (42.3)	17 (47.2)	
Postmenopause	79	60 (57.7)	19 (52.8)	
Family history of breast cancer				1.000
No	133	99 (95.2)	34 (94.4)	
Yes	7	5 (4.8)	2 (5.6)	

BMI: body mass index.

### 3.2 B-mode and CEUS characteristics

**Figure 3:**
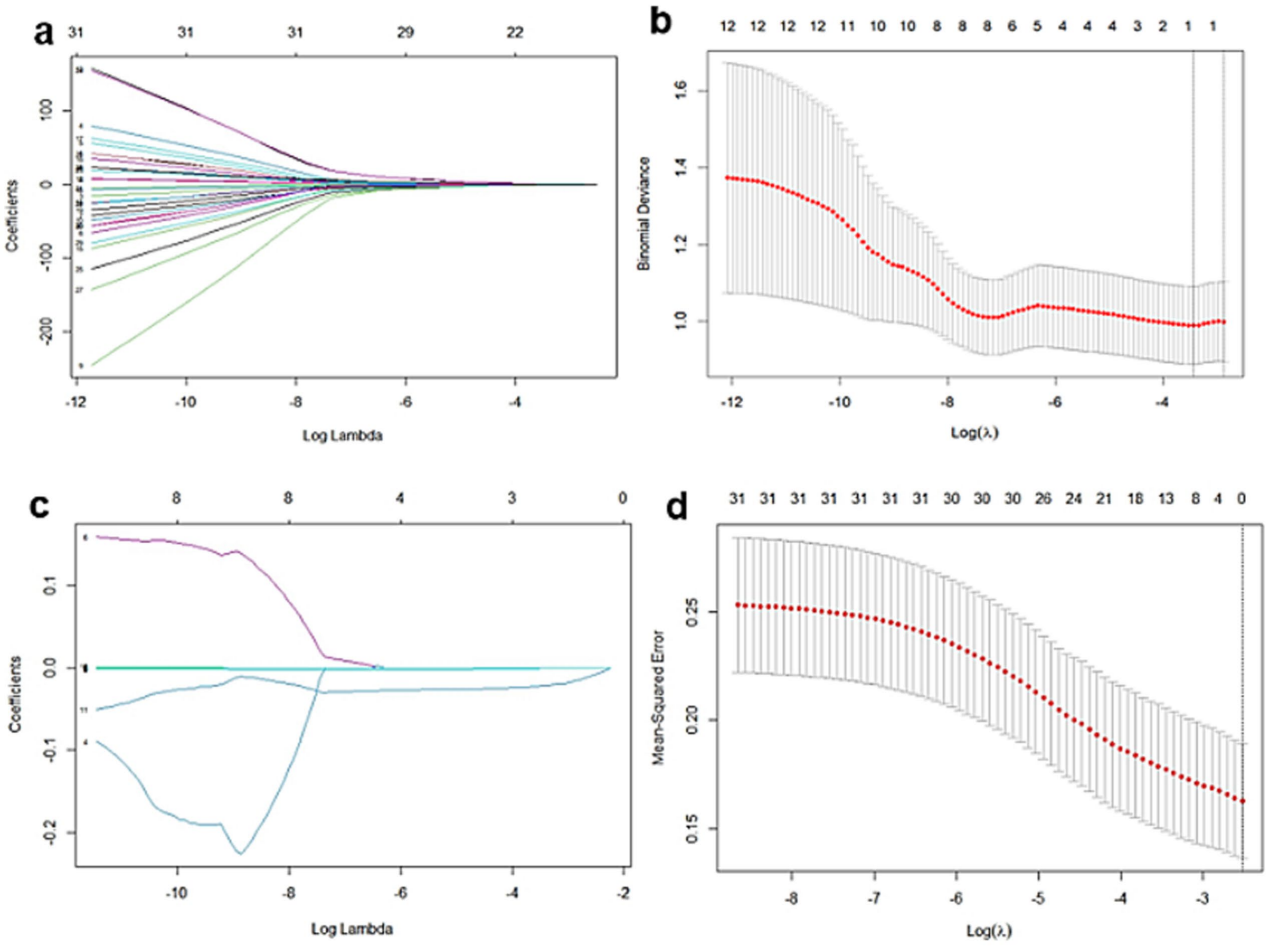
Feature selection in B-mode, CEUS, and TIC of the CEUS group by LASSO regression in 140 patients with breast cancer. **(a,b)** Selection of B-mode ultrasound and CEUS features. **(c,d)** Selection of TIC parameters.

In 140 patients with breast cancer, the image features of B-mode ultrasound, CEUS, and TIC were assessed (Supplementary Table 2). According to the LASSO regression in clinical B-mode with CEUS and TIC of CEUS characteristic groups, seven features related to HER-2 positive breast cancer, including tumor size (cm), echotexture, strip-shaped echoic, macrocalcifications, microcalcifications, perfusion defects, and fall time (FT) of TIC, were selected (Figure 3). No clinical characteristics were observed. The distribution of the selected characteristics is listed in Table 2.

**Table 2:** Selected features of 140 patients in training and validation sets.

	Validation set (*n* = 36) (%)	Training set (*n* = 104) (%)	Total (*n* = 140)
Tumor size (cm)	2.45 ± 1.18	2.11 ± 1.05	2.20 ± 1.09 (0.5–5.9)
Echotexture			
Homogeneous	11 (30.6)	26 (25.0)	37
Heterogeneous	25 (69.4)	78 (75.0)	103
Strip-shaped echoic			
Absence	11 (30.6)	32 (30.8)	43
Present	25 (69.4)	72 (69.2)	97
Macrocalcifications			
Absence	7 (19.4)	14 (13.5)	21
Present	29 (80.6)	90 (86.5)	119
Microcalcifications			
Absence	31 (86.1)	71 (68.3)	102
Present	5 (13.9)	33 (31.7)	38
Perfusion defects			
Presence	13 (36.1)	34 (32.7)	47
Absence	23 (63.9)	70 (67.3)	93
FT (s)	17.45 ± 18.46	17.49 ± 13.33	-

CEUS: contrast-enhanced ultrasound; TIC: time intensity curve; FT: fall time.

Characteristics of B-mode imaging with CEUS.


$$\begin{array}{c} 0.19952497+[0.01610785*\text{Tumor size}]\\ +[0.01815141*\text{Echotexture}]\\ +[-0.07019562*\text{Strip}-\text{shaped echoic}]\\ +[-0.01206527*\text{Macrocalcifications}]\\ +[0.04789061*\text{Microcalcifications}]\\ +[-0.01622952*\text{Perfusion defect}]. \end{array}$$

Characteristics of CEUS TIC.


−1.21674647+[−0.01299429∗FT].

In 107 cases in the HER2 low expression group, the image features of the three modalities were assessed in Supplementary Table 3. Imaging features related to HER2 low expression were selected by LASSO regression, including location, shape, strip-shaped echoic, perfusion defect, mean transit time (mTT), and FT. There were no clinical characteristics observed. The selected characteristics are listed in Supplementary Table 4.

Characteristics in B-mode.


$$\begin{array}{l} 0.13508836+[0.16800326*\text{Location}]+[0.31750333*\text{Shape}]\\ +[0.02871666*\text{Strip}-\text{shaped echoic}]. \end{array}$$

Characteristics of CEUS images.


1.3810282+[−0.6861225∗Perfusion defect].

Characteristics in TIC of CEUS.


1.2891179887+[−0.0002615468∗mTT]+[−0.0197138051∗FT].

### 3.3 Machine learning models for the prediction

The prediction model was established on the training set, and its performance was tested on the validation set. The effectiveness and stability of the training set, consisting of 104 cases, were validated using LOOCV, and the accuracy and Kappa were 0.871 and 0.446, respectively. In the training set of FISH positive (IHC 3+) and negative groups, six classifiers, including logistic regression (LR), support vector machine (SVM), random forest (RF), XGB (XGBoost), decision-level fusion technique of hard voting based on LR, SVM, and XGB, as well as the XGB combined with the LR model (29, 30).

**Figure 4:**
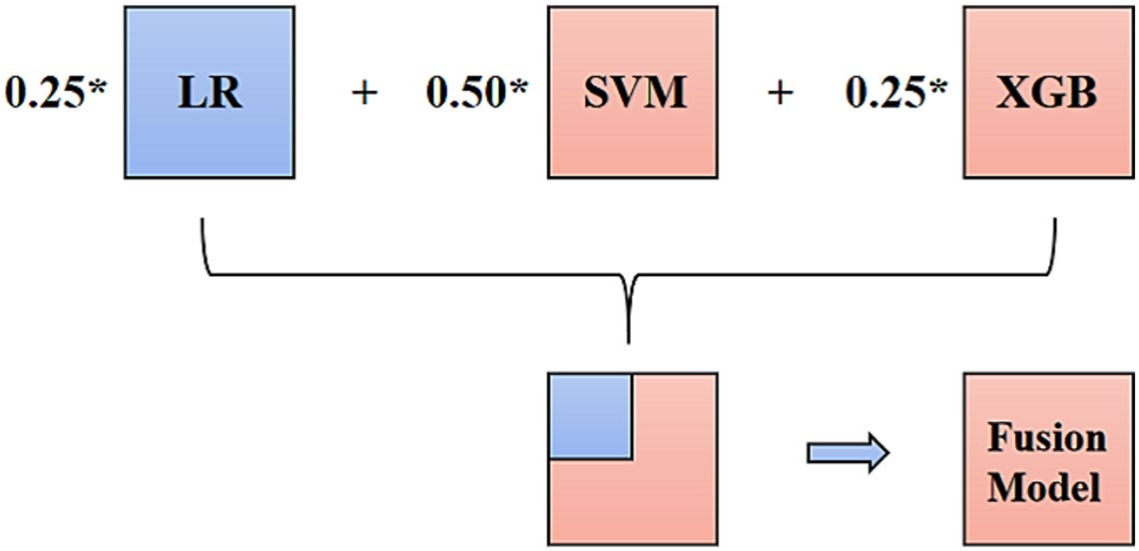
Hard voting progression of the decision-level fusion model.

The final result of the decision-level fusion model was determined by three single classifiers: LR, SVM, and XGB (better than RF in this study). The hard-voting progression is shown in Figure 4. In the XGB combined with LR prediction model, XGB was used to construct new variables, reflecting the correlation of the selected variables. LR was used to gather the selected and new variables to construct the prediction model and to calculate the significance and weight coefficients of each variable. In the prediction of the HER2-positive breast cancer group, seven variables, including a novel feature (V11) generated by the XGB tree-based model trained on existing features, were selected for the final LR prediction model based on the feature importance rankings (Supplementary Figure 1).

Classifiers of LR, SVM, RF, and XGB were established in three imaging modalities: (1) B-mode ultrasound, (2) B-mode ultrasound combined with CEUS, and (3) B-mode ultrasound combined with CEUS and TIC. The other two types of fusion models were used in the third multi-modality to predict HER2-positive breast cancer.

The AUC, sensitivity, specificity, and accuracy of the four classifiers in three modalities are shown in Table 3. The sensitivities of SVM were increased from 0.728 (95%CI: 0.554–0.862) to 0.778 (95%CI: 0.608–0.899) by adding the CEUS modality. In the three modalities group, SVM performs the best AUC value in the four single classifiers, with an AUC of 0.806 (95%CI: 0.640–0.918), a sensitivity of 0.833 (95%CI: 0.359–0.996) and a specificity of 0.767 (95%CI: 0.577–0.901). The AUC values improved with the enrichment of the imaging modalities. In the third modality, the performances of the other two fusion models are also shown in Table 3.

**Table 3:** Diagnostic performance of the classifiers in predicting HER2-positive patients.

	AUC (95%CI)	Sensitivity (95%CI)	Specificity (95%CI)	Accuracy
B-mode ultrasound				
RF	0.567 (0.392–0.730)	0.167 (0.004–0.641)	0.967 (0.828–0.999)	0.833
B-mode ultrasound and CEUS				
SVM	0.778 (0.608–0.899)	0.667 (0.223–0.957)	0.867 (0.693–0.962)	0.833
RF	0.583 (0.408–0.745)	0.167 (0.004–0.641)	1.000 (0.884–1.000)	0.861
B-mode ultrasound, CEUS, and TIC				
LR	0.633 (0.457–0.787)	0.667 (0.223–0.957)	0.767 (0.577–0.901)	0.722
SVM	0.806 (0.640–0.918)	0.833 (0.359–0.996)	0.767 (0.577–0.901)	0.778
RF	0.583 (0.408–0.745)	0.167 (0.400–0.641)	1.000 (0.884–1.000)	0.861
XGB	0.700 (0.525–0.841)	0.500 (0.118–0.882)	0.900 (0.735–0.979)	0.833
XGB + LR	0.689 (0.513–0.832)	0.668 (0.223–0.957)	0.633 (0.439–0.801)	0.639
LR + SVM + XGB	0.869 (0.715–0.958)	1.000 (0.541–1.000)	0.668 (0.472–0.827)	0.722

CEUS: contrast-enhanced ultrasound; TIC: time intensity curve; LR: logistic regression; SVM: support vector machine; RF: random forest; XGB: XGBoost.

**Figure 5:**
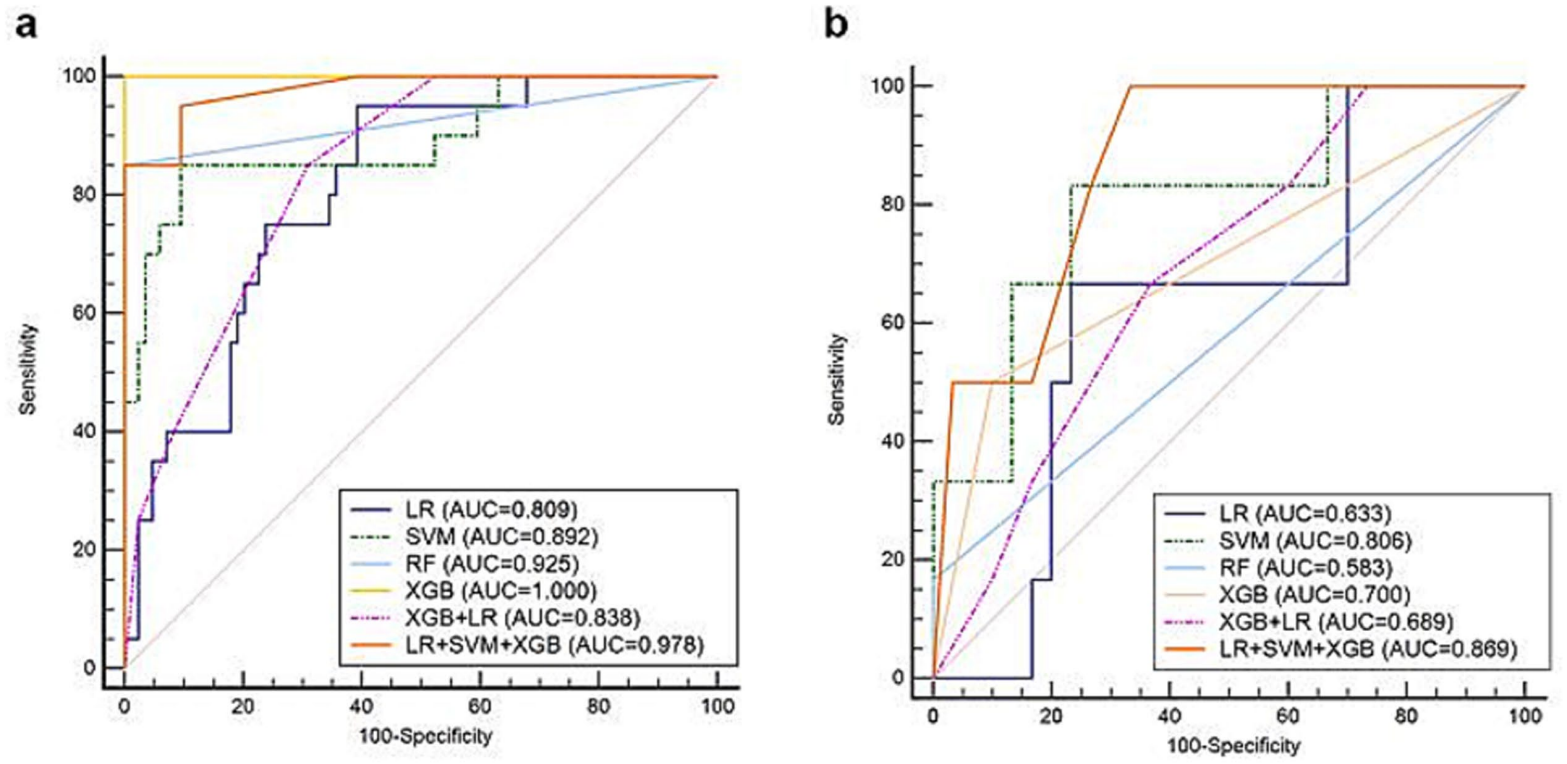
ROCs of the classifiers in predicting HER2-positive breast cancer based on B-mode ultrasound, CEUS, and TIC in the **(a)** training and **(b)** validation sets.

According to the predictive performance of LR, SVM, RF, and XGB, the three top-performing individual classifiers for HER2 expression, LR, SVM, and XGB, were combined using hard voting to generate a consolidated prediction result. Thus, the decision-level fusion model was constructed using hard voting based on LR, SVM, and XGB to establish the fusion model, and the weighted ratio was set as 1:1:1. In the six models, the fusion model of LR, SVM, and XGB classifiers performed best, with an AUC value of 0.869 (95%CI: 0.715–0.958), a sensitivity of 1.000 (95%CI: 0.541–1.000), and a specificity of 0.668 (95%CI: 0.472–0.827). The ROCs of the six classifiers in B-mode ultrasound combined with CEUS and TIC modalities are shown in Figure 5. In the training cohort of 104 cases, 31 cases with certain IHC results were assessed as IHC 2 + by CNB, and two of them were reclassified as IHC 3 + according to FISH results. The fusion model of LR, SVM, and XGB also predicted them as IHC 3 + cases.

**Figure 6:**
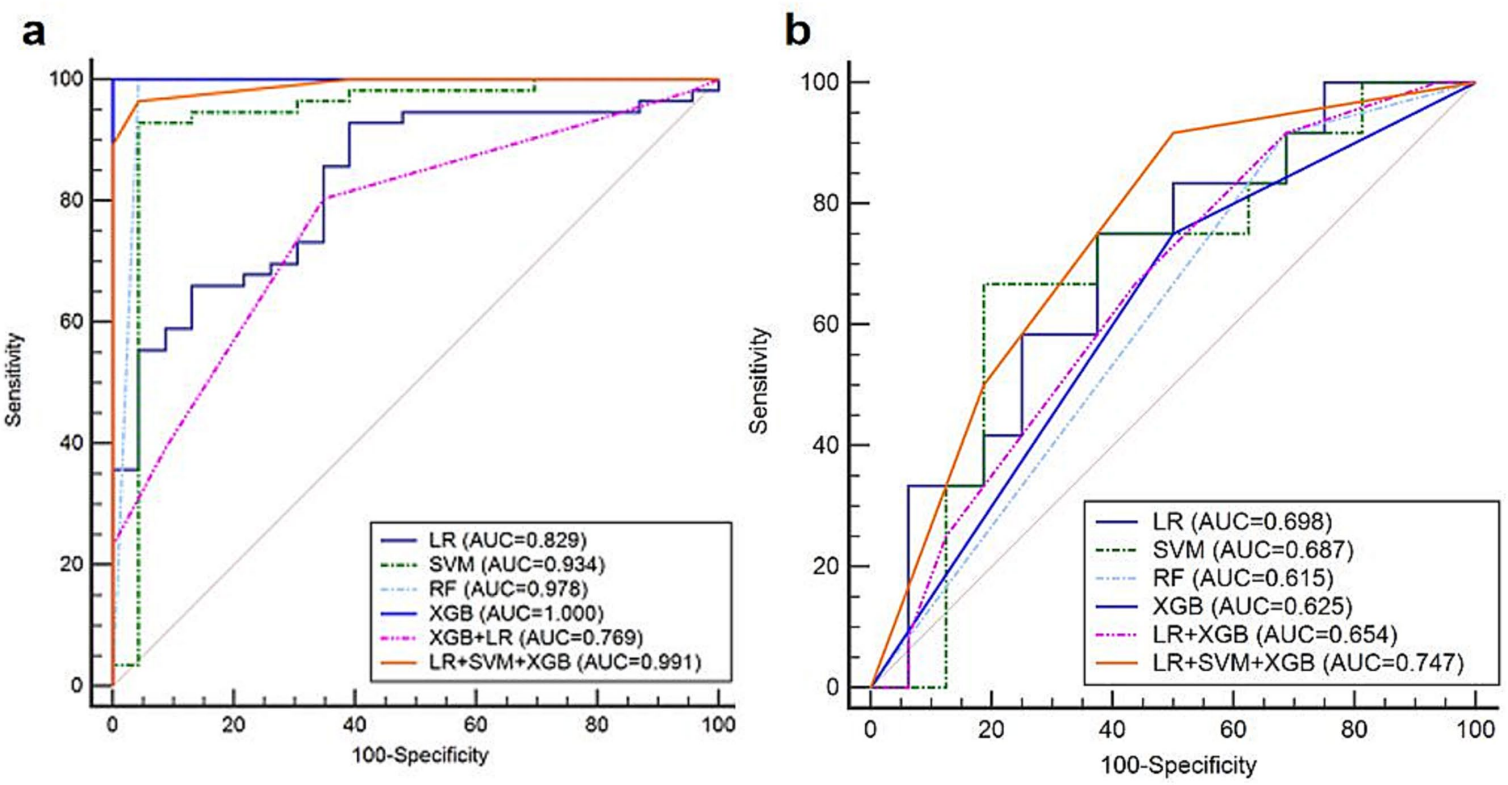
The ROCs of the six prediction models in predicting HER2 low expression patients in the **(a)** training and **(b)** validation sets.

In the training set of the HER2 low expression and HER2-negative groups, prediction models based on the six classifiers in the third modality were also established. In the training set of 79 participants, the accuracy and kappa values were 0.864 and 0.637, respectively. The AUC values, sensitivity, specificity, and accuracy are shown in Table 4. The decision-level fusion model was selected as the voting result of LR, SVM, and XGB, and the weighted ratio was set at 1:2:1, according to the performance of the classifiers. The fusion model of LR, SVM, RF, and XGB classifiers also gets the highest AUC value of 0.747 (95%CI: 0.548–0.891), sensitivity of 1.000 (95%CI: 0.735–1.000), and specificity of 0.438 (95%CI: 0.198–0.701). The ROCs of the six prediction models in the HER2 low expression and negative group are shown in Figure 6. Both the AUCs for predicting HER2 status were increased using the decision-level machine learning approach.

**Table 4:** Diagnostic performance of the classifiers in predicting HER2 low expression patients based on B-mode ultrasound, CEUS, and TIC characteristics.

Models	AUC (95%CI)	Sensitivity (95%CI)	Specificity (95%CI)	Accuracy
LR	0.698 (0.496–0.856)	0.750 (0.428–0.945)	0.625 (0.354–0.848)	0.679
SVM	0.687 (0.486–0.848)	0.667 (0.349–0.901)	0.813 (0.544–0.960)	0.750
RF	0.615 (0.413–0.791)	0.917 (0.615–0.998)	0.313 (0.110–0.587)	0.571
XGB	0.625 (0.423–0.799)	0.750 (0.428–0. 945)	0.500 (0.247–0.753)	0.607
XGB + LR	0.654 (0.451–0.822)	0.917 (0.615–0.998)	0.313 (0.110–0.587)	0.571
LR + SVM + XGB	0.747 (0.548–0.891)	0.917 (0.615–0.998)	0.500 (0.247–0.753)	0.679

AUC, area under curve; CI, confidence interval; LR: logistic regression; SVM: support vector machine; RF: random forest; XGB: XGBoost.

## 4 Discussion

### 4.1 Key findings in the context of prior literature

HER2-targeted therapy can reduce recurrence and increase the likelihood of breast-conserving surgery in patients with HER2-positive breast cancer. In this study, the fusion model of multiple single classifiers, based on machine learning approaches, performed best in predicting HER2 3 + and HER2 2+/1 + expression, with an AUC of 0.869 (95%CI: 0.715–0.958) and 0.747 (95%CI: 0.548–0.891), respectively. It could also predict the two equivocal IHC 2 + breast cancers as HER2 3+, in concordance with the FISH results.

In this research, imaging features of multimodalities, including B-mode ultrasound, CEUS, and TIC, were obtained by assessment of radiologists. Previous studies that predicted HER2 expression using imaging features are shown in Supplementary Table 5. Compared with radiomic features acquired by software or a single ultrasound modality, these features are more available and can provide abundant vascularity information. Vasculogenic mimicry (VM), which differs from angiogenesis formed by endothelial cells, is a vascular structure formed by cancer cells that transit tumor and blood cells in a channel network and is involved in tumor neovascularization (31, 32). In breast cancer, VM is associated with HER2-positive cases, which may contribute to two anticoagulant-secreted proteins, Serpine2 and Slpi, promoting VM formation. Both of them mostly occurred in HER2-positive patients with breast cancer (33, 34). Studies have shown that CEUS can assess VM density *in vitro*, and the quantitative parameters of TIC are related to VM (35, 36). Thus, the microbubbles of CEUS may provide information on HER2-positive breast cancer neovascularization at the molecular level.

Previous studies have mostly focused on HER2 3 + expression in breast cancers using radiomic approaches. To the best of our knowledge, this is the first study to use LR, SVM, and XGB fusion models by voting decision method to prospectively predict HER2 3 + and 2+/1 + expression levels in breast cancer based on a multicenter study of contrast Sonazoid-enhanced ultrasonography. In predicting HER2-positive and HER2-low expression BC cases, the AUC values of the fusion model in both of the two groups were the highest compared with other single machine learning models.

### 4.2 Clinical implications and innovations

In this study, tumor size, echotexture, strip-shaped echoic, macrocalcifications, and microcalcifications on B-mode ultrasound, perfusion defects on CEUS, and FT of TIC were predictive factors of HER2-positive breast cancer. Factors including tumor location, shape, strip-shaped echoic in B-mode ultrasound, perfusion defect in CEUS, mTT, and FT of TIC could predict HER2 low expression. Strip-shaped echogenic perfusion defects and FT are also predictors of HER2-positive expression, indicating that these features may be closely related to HER2 protein expression levels (2, 37).

Tumor size may reflect growth, indicating the prognosis of malignant tumors. Features of macrocalcifications and microcalcifications on B-mode ultrasound were associated with HER2-positive breast cancer in this study, which was also consistent with previous studies (38–41). Macrocalcification is regarded as the degeneration of the breast caused by injury and inflammation unrelated to cancer, while microcalcification is regarded as a calcium spot caused by rapid decomposition of cancer cells (38). With high aggressiveness and poor prognosis, HER2-positive breast cancer may be related to a faster growth rate than negative cases, indicating that more cell decomposition of the breast exists in positive cases (42, 43).

A strip-shaped echo mostly indicates the fibrosis inside the tumor. Malignant lesions can exhibit disordered hyperechoic strands, whereas benign lesions tend to exhibit organized linear echoes. Fibrosis in breast tumors is histologically regarded as fibroblasts and collagen fibers in the tumor center (44). Some studies have reported that fibrosis is positively related to HER2 expression and high aggressiveness of tumors (45), which is in contrast to the results of this study. In this study, fewer strip-shaped echoes were observed in HER2-positive breast cancer. A possible reason may be that most of our breast cancer cases were in stage I or II (100/104), and tumor cells were in the rapid growth phase, without undergoing necrosis and fibrosis. Further studies are still needed to determine the relationship between strip-shaped echoes and HER2 expression (45, 46).

Previous studies have also revealed that high HER2 expression might be related to the increased invasiveness of tumor cells and the formation of neovasculature (47). In some studies, perfusion defects in CEUS more frequently occurred in HER2-positive breast cancer, which might be caused by ischemic necrosis of the tumor, contributing to the slower blood vessel growth rate than the increased oxygen consumption of the tumor cells (48, 49). Other studies have also revealed that perfusion defects might be associated with uneven distribution of the contrast agent caused by heterogeneity and blood vessel distribution inside the tumor (47, 50). However, in this study, perfusion defects in Sonazoid-based CEUS were negatively associated with HER2-positive and low-expression breast cancers. In HER2 expression cases, less fibrosis was observed, indicating the presence of abundant vascularity, compared with HER2-negative cases.

In SVM models of three modalities, the sensitivities in predicting HER2-positive breast cancer were increased by CEUS, from 0.728 (95%CI: 0.554–0.862) to 0.778 (95%CI: 0.608–0.899). By adding the TIC feature, the sensitivity could also be increased, up to 0.806 (95%CI: 0.640–0.918). This result may indicate that the evaluation of microvasculature could improve the performance of prediction models in HER2-positive breast cancer, especially for the evaluation of TIC features. In previous studies of Sonazoid-based CEUS in liver cancer, short mTT and FT could be factors that differentiate angiomyolipoma and hepatocellular carcinoma from hepatocellular carcinoma because of the different amounts of blood vessels (51). In this study, short FT may be associated with HER2 expression (IHC3+, 2+, and 1+) in breast cancer, compared with HER2-negative expression cases. This may be related to the rapid excretion rate of Sonazoid microbubbles from intratumoral vessels in HER2 expression breast lesions. FT may be related to the number of blood vessels inside tumors because abundant vessels may contribute to a fast blood flow discharging from the draining vein and a short contrast agent staying time. Therefore, HER2-expressing breast tumors tend to exhibit higher internal vascularity.

In the 104 cases of patients with breast cancer, there were a total of 31 cases defined as IHC 2 + for the first time of CNB, with certain results of biopsy. Two of these were finally defined as IHC 3 + according to the FISH results, revealing that 6.5% (2/31) of HER2-positive cases were underestimated by IHC in this study. In the prediction results of the LR + SVM + XGB fusion model, the two cases were also predicted as IHC 3+, indicating that the fusion predictive model could improve the detection of IHC 3 + compared with the results of CNB by pathologists.

### 4.3 Limitations and future directions

Our study used LR, SVM, and XGB decision-level fusion models to predict three HER2 expression levels in breast cancer in two cohorts based on a prospective multicenter study of contrast Sonazoid-enhanced and B-mode ultrasound. However, this study has some limitations. First, the number of cases was limited because of the use of Sonazoid in breast CEUS multicenter studies. Second, this study only contained image features evaluated by radiologists. Radiomic features can reflect unrecognizable and quantifiable messages to the naked eye. Using radiomic approaches in multi-modal ultrasound may improve the prediction of BC biomarkers. However, radiomic features extracted by software were less available compared with the features assessed by radiologists in this study. Third, our study only included images from B-mode ultrasound and CEUS. Additional modalities, such as MRI and mammography, are expected to be included in the prediction of HER2 expression.

## 5 Conclusion

In conclusion, multi-mode ultrasound, including B-mode ultrasound, CEUS, and TIC, can predict HER2 expression status. Moreover, the fusion model of machine learning classifiers can improve the prediction results.

## Data Availability

The raw data supporting the conclusions of this article will be made available by the authors, without undue reservation.
