# Supplementary material for: Machine learning-based fusion model for predicting HER2 expression in breast cancer by Sonazoid-enhanced ultrasound: a multicenter study
Source: Front Med (Lausanne). 2025 May 21;12:1585823. doi: 10.3389/fmed.2025.1585823 (PMC12133895; doi:10.3389/fmed.2025.1585823)
Supplement: Supplementary file 1 [file Data_Sheet_1.doc]

**Supplementary materials**

**Supplementary table 1.** Ultrasound devices and linear probes.

| **Manufacturer** | **System** | **Country** | **Probe settings** |
| --- | --- | --- | --- |
| Conon/TOSHIBA | Aplio 500 | Japan | 14L5 |
| Conon/TOSHIBA | Aplio i800 | Japan | i18LX5 |
| GE | LOGIQ e9 | American | ML 6-15 |
| GE | LOGIQ e9 | American | 9L |
| GE | LOGIQ e20 | American | 9L |
| Hitachi | Aloka | Japan | L441 |
| Mindray | Resona7 | China | L9-3U |
| Philips | IU-22 | American | L12-5 |
| Philips | IE33 | American | L12-5 |
| Philips | EPIQ7 | American | eL18-4 |
| SAMSUNG | RS80A | Korea | LA2-9A |
| SAMSUNG | RS85 | Korea | LA2-9A |
| Supersonic | Aixplorer | French | SL10-2 |
| Supersonic | Aixplorer V | French | SL10-2 |
| Siemens | Sequaia | Germany | 9L |

**Supplementary table 2.** Imaging characteristics in three modalities in the training and validation set of HER2 0, 1+, 2+ or 3+ breast cancer patients.

|  | **Training set (n=104) (%)** | **Validation set (n=36) (%)** | **Total (n=140)** | **p value** |
| --- | --- | --- | --- | --- |
| **Clinical characteristics** | | | | |
| Age (years) | 50.11 ± 10.75 | 53.13 ± 11.07 | 52.35 ± 11.03 (23-85) | 0.158 |
| BMI (kg/m^2^) | 23.46 ± 4.18 | 24.46 ± 5.30 | 24.20 ± 5.04 (13.65-63.70) | 0.308 |
| Menopause |  |  |  | 0.608 |
| Premenopause | 17 (47.2) | 44 (42.3) | 61 |  |
| Postmenopause | 19 (52.8) | 60 (57.7) | 79 |  |
| Family history of breast cancer |  |  |  | 1.000 |
| No | 34 (94.4) | 99 (95.2) | 133 |  |
| Yes | 2 (5.6) | 5 (4.8) | 7 |  |
| **B-mode ultrasound characteristics** | | | | |
| Tumor size (cm) | 2.11±1.05 | 2.45 ± 1.18 | 2.20 ± 1.09 (0.5-5.9) | 0.105 |
| Location |  |  |  | 0.044 |
| Upper outer quadrant | 34 (32.7) | 14 (38.9) | 48 |  |
| Upper inner quadrant | 14 (13.5) | 2 (5.6) | 16 |  |
| Lower outer quadrant | 6 (5.8) | 0 (0) | 6 |  |
| Lower inner quadrant | 25 (24.0) | 16 (44.4) | 41 |  |
| Others | 25 (24.0) | 4 (11.1) | 29 |  |
| Margin |  |  |  | 0.177 |
| Circumscribed | 28 (26.9) | 14 (38.9) | 42 |  |
| Uncircumscribed | 76 (73.1) | 22 (61.1) | 98 |  |
| Cub foot sign |  |  |  | 0.681 |
| No | 59 (56.7) | 19 (52.8) | 78 |  |
| Yes | 45 (43.3) | 17 (47.2) | 62 |  |
| Shape |  |  |  | 0.028 |
| Regular | 19 (18.3) | 13 (36.1) | 32 |  |
| Irregular | 85 (81.7) | 23 (63.9) | 108 |  |
| Orientation |  |  |  | 0.194 |
| Parallel | 86 (82.7) | 33 (91.7) | 119 |  |
| Vertical | 18 (17.3) | 3 (8.3) | 21 |  |
| Boundary |  |  |  | 0.313 |
| Clear | 18 (17.3) | 9 (25.0) | 27 |  |
| Unclear | 86 (82.7) | 27 (75.0) | 113 |  |
| Capsule |  |  |  | 0.280 |
| Absence | 6 (5.8) | 4 (11.1) | 10 |  |
| Present | 98 (94.2) | 32 (88.9) | 130 |  |
| Hypoechoic halo |  |  |  | 0.557 |
| No | 11 (10.6) | 5 (13.9) | 16 |  |
| Yes | 93 (89.4) | 31 (86.1) | 124 |  |
| Hyperechoic halo |  |  |  | 0.173 |
| No | 54 (51.9) | 24 (66.7) | 78 |  |
| Yes | 50 (48.1) | 12 (33.3) | 62 |  |
| Echogenicity |  |  |  | 0.445 |
| Hypoechoic | 86 (82.7) | 32 (88.9) | 118 |  |
| Isoechoic | 4 (3.8) | 0 (0) | 4 |  |
| Mix echoic | 14 (13.5) | 4 (11.1) | 18 |  |
| Echotexture |  |  |  | 0.518 |
| Homogeneous | 26 (25.0) | 11 (30.6) | 37 |  |
| Heterogeneous | 78 (75.0) | 25 (69.4) | 103 |  |
| Posterior features |  |  |  | 0.418 |
| Shadowing | 31 (29.8) | 11 (30.6) | 42 |  |
| No posterior features | 55 (52.9) | 15 (41.7) | 70 |  |
| Enhancement | 17 (16.3) | 10 (27.8) | 27 |  |
| Combined pattern | 1 (1.0) | 0 (0) | 1 |  |
| Strip-shaped echoic |  |  |  | 0.981 |
| Absence | 32 (30.8) | 11 (30.6) | 43 |  |
| Present | 72 (69.2) | 25 (69.4) | 97 |  |
| Macrocalcifications |  |  |  | 0.386 |
| Absence | 14 (13.5) | 7 (19.4) | 21 |  |
| Present | 90 (86.5) | 29 (80.6) | 119 |  |
| Microcalcifications |  |  |  | 0.038 |
| Absence | 71 (68.3) | 31 (86.1) | 102 |  |
| Present | 33 (31.7) | 5 (13.9) | 38 |  |
| Nourish vessel |  |  |  | 0.734 |
| Absence | 40 (38.5) | 15 (41.7) | 55 |  |
| Present | 64 (61.5) | 21 (58.3) | 85 |  |
| Blood flow grade |  |  |  | 0.726 |
| Grade 0 | 14 (13.5) | 6 (16.7) | 20 |  |
| Grade 1 | 47 (45.2) | 14 (38.9) | 61 |  |
| Grade 2 | 27 (26.0) | 8 (22.2) | 35 |  |
| Grade 3 | 16 (15.4) | 8 (22.2) | 24 |  |
| **CEUS characteristics** | | | | |
| Wash-in time |  |  |  | 0.174 |
| Earlier | 89 (85.6) | 33 (91.7) | 122 |  |
| Later | 9 (8.7) | 0 (0) | 9 |  |
| Synchronous | 6 (5.8) | 3 (8.3) | 9 |  |
| Enhancement degree |  |  |  | 0.143 |
| Hyperenhancement | 85 (81.7) | 34 (94.4) | 119 |  |
| Isoenhancement | 7 (6.7) | 0 (0) | 7 |  |
| Hypoenhance | 12 (11.5) | 2 (5.6) | 14 |  |
| Entirely wash-out time of lesions |  |  |  | 0.963 |
| ≤ 5mins | 38 (36.5) | 13 (36.1) | 51 |  |
| > 5mins | 66 (63.5) | 23 (63.9) | 89 |  |
| Uptake pattern |  |  |  | 0.442 |
| Centripetal | 58 (55.8) | 16 (44.4) | 74 |  |
| Centrifugal | 2 (1.9) | 2 (5.6) | 4 |  |
| Diffuse | 43 (41.3) | 18 (50.0) | 61 |  |
| No enhance | 1 (1.0) | 0 (0) | 1 |  |
| Homogeneous pattern |  |  |  | 0.158 |
| Presence | 20 (19.2) | 11 (30.6) | 31 |  |
| Absence | 84 (80.8) | 25 (69.4) | 109 |  |
| Rim like enhancement |  |  |  | 0.453 |
| Presence | 95 (91.3) | 35 (97.2) | 130 |  |
| Absence | 9 (8.7) | 1 (2.8) | 10 |  |
| Claw shaped pattern |  |  |  | 0.120 |
| Presence | 63 (60.6) | 27 (75.0) | 90 |  |
| Absence | 41 (39.4) | 9 (25.0) | 50 |  |
| Perfusion defects |  |  |  | 0.708 |
| Presence | 34 (32.7) | 13 (36.1) | 47 |  |
| Absence | 70 (67.3) | 23 (63.9) | 93 |  |
| Lesion size compared with conventional ultrasound increased |  |  |  | 0.632 |
| Presence | 22 (21.2) | 9 (25.0) | 31 |  |
| Absence | 82 (78.8) | 27 (75.0) | 109 |  |
| Margin |  |  |  | 0.389 |
| Well defined | 34 (32.7) | 9 (25.0) | 43 |  |
| Poorly defined | 70 (67.3) | 27 (75.0) | 97 |  |
| Shape |  |  |  | 0.087 |
| Regular | 25 (24.0) | 14 (38.9) | 39 |  |
| Irregular | 79 (76.0) | 22 (61.1) | 101 |  |
| Nourishing vessels |  |  |  | 0.737 |
| Presence | 29 (27.9) | 9 (25.0) | 38 |  |
| Absence | 75 (72.1) | 27 (75.0) | 102 |  |
| **TIC parameters** | | | | |
| MeanLin | 12221.75 ± 75726.67 | 278643.88 ± 844406.68 | - | 0.067 |
| PE | 66138.21 ± 459696.33 | 1320696.66 ± 4043742.51 | - | 0.072 |
| RT (s) | 6.89 ± 4.24 | 6.99 ± 4.37 | - | 0.911 |
| TTP (s) | 9.73 ± 6.11 | 10.50 ± 6.29 | - | 0.519 |
| mTT (s) | 75.19 ± 57.87 | 64.15 ± 46.21 | - | 0.302 |
| FT (s) | 17.49 ± 13.33 | 17.45 ± 18.46 | - | 0.987 |
| WiAUC | 149815.27 ± 880953.59 | 3567242.86 ± 10092094.80 | - | 0.050 |
| WiR | 29153.17 ± 228013.70 | 462203.47 ± 1478986.62 | - | 0.089 |
| WiPI | 42340.14 ± 295489.06 | 837389.64 ± 2561468.63 | - | 0.072 |
| WoAUC | 286549.42 ± 1703846.82 | 6996277.89 ± 19239424.10 | - | 0.044 |
| WoR | 10874.87 ± 81120.19 | 184767.13 ± 572466.47 | - | 0.078 |
| WiWoAUC | 436461.31 ± 2587617.34 | 10557812.58 ± 29307857.56 | - | 0.046 |

Note: CEUS: contrast enhanced ultrasound; MeanLin: the average contrast signal intensity; PE: peak enhancement; RT: rising time; TTP: time to peak; mTT: mean transit time; FT: fall time; WiAUC: Wash-in Area Under the Curve; WiR: Wash-in Rate; WiPI: Wash-in Perfusion Index; WoAUC: Wash-out Area Under the Curve; WoR: Wash-out Rate; WiWoAUC: WiAUC + WoAUC.

**Supplementary table 3.** Imaging characteristics in three modalities in the training and validation set of HER2 0, 1+ or 2+ breast cancer patients.

|  | **Training set (n=79) (%)** | **Validation set (n=28) (%)** | **Total (n=107)** | **p value** |
| --- | --- | --- | --- | --- |
| **B-mode ultrasound characteristics** | | | | |
| Tumor size (cm) | 2.36±1.26 | 2.05±1.07 | 2.13±1.12 | 0.201 |
| Location |  |  |  | 0.157 |
| Upper outer quadrant | 25 (31.6) | 11 (39.3) | 36 |  |
| Upper inner quadrant | 11 (13.9) | 1 (3.6) | 12 |  |
| Lower outer quadrant | 6 (7.6) | 0 (0) | 6 |  |
| Lower inner quadrant | 21 (26.6) | 12 (42.9) | 33 |  |
| Others | 16 (20.3) | 4 (14.3) | 20 |  |
| Margin |  |  |  | 0.038 |
| Circumscribed | 20 (25.3) | 13 (46.4) | 33 |  |
| Uncircumscribed | 59 (74.7) | 15 (53.6) | 74 |  |
| Cub foot sign |  |  |  | 0.730 |
| No | 45 (57.0) | 17 (60.7) | 62 |  |
| Yes | 34 (43.0) | 11 (39.3) | 45 |  |
| Shape |  |  |  | 0.005 |
| Regular | 13 (16.5) | 12 (42.9) | 25 |  |
| Irregular | 66 (83.5) | 16 (57.1) | 82 |  |
| Orientation |  |  |  | 0.108 |
| Parallel | 66 (83.5) | 27 (96.4) | 93 |  |
| Vertical | 13 (16.5) | 1 (3.6) | 14 |  |
| Boundary |  |  |  | 0.092 |
| Clear | 11 (13.9) | 8 (28.6) | 19 |  |
| Unclear | 68 (86.1) | 20 (71.4) | 88 |  |
| Capsule |  |  |  | 0.202 |
| Absence | 4 (5.1) | 4 (14.3) | 8 |  |
| Present | 75 (94.9) | 24 (85.7) | 99 |  |
| Hypoechoic halo |  |  |  | 0.285 |
| No | 6 (7.6) | 4 (14.3) | 10 |  |
| Yes | 73 (92.4) | 24 (85.7) | 97 |  |
| Hyperechoic halo |  |  |  | 0.044 |
| No | 42 (53.2) | 21 (75.0) | 63 |  |
| Yes | 37 (46.8) | 7 (25.0) | 44 |  |
| Echogenicity |  |  |  | 0.475 |
| Hypoechoic | 65 (82.3) | 24 (85.7) | 89 |  |
| Isoechoic | 4 (5.1) | 0 (0) | 4 |  |
| Mix echoic | 10 (12.7) | 4 (14.3) | 14 |  |
| Echotexture |  |  |  | 0.764 |
| Homogeneous | 23 (29.1) | 9 (32.1) | 32 |  |
| Heterogeneous | 56 (70.9) | 19 (67.9) | 75 |  |
| Posterior features |  |  |  | 0.411 |
| Shadowing | 23 (29.1) | 8 (28.6) | 31 |  |
| No posterior features | 43 (54.4) | 12 (42.9) | 55 |  |
| Enhancement | 12 (15.2) | 8 (28.6) | 20 |  |
| Combined pattern | 1 (1.3) | 0 (0) | 1 |  |
| Strip-shaped echoic |  |  |  | 0.485 |
| Absence | 20 (25.3) | 9 (32.1) | 29 |  |
| Present | 59 (74.7) | 19 (67.9) | 78 |  |
| Macrocalcifications |  |  |  | 0.772 |
| Absence | 7 (8.9) | 3 (10.7) | 10 |  |
| Present | 72 (91.1) | 25 (89.3) | 97 |  |
| Microcalcifications |  |  |  | 0.229 |
| Absence | 59 (74.7) | 24 (85.7) | 83 |  |
| Present | 20 (25.3) | 4 (14.3) | 24 |  |
| Nourish vessel |  |  |  | 0.737 |
| Absence | 31 (39.2) | 12 (42.9) | 43 |  |
| Present | 48 (60.8) | 16 (57.1) | 64 |  |
| Blood flow grade |  |  |  | 0.510 |
| Grade 0 | 9 (11.4) | 5 (17.9) | 14 |  |
| Grade 1 | 38 (48.1) | 9 (32.1) | 47 |  |
| Grade 2 | 19 (24.1) | 8 (28.6) | 27 |  |
| Grade 3 | 13 (16.5) | 6 (21.4) | 19 |  |
| **CEUS characteristics** | | | | |
| Wash-in time |  |  |  | 0.260 |
| Earlier | 66 (83.5) | 26 (92.9) | 92 |  |
| Later | 7 (8.9) | 0 (0) | 7 |  |
| Synchronous | 6 (7.6) | 2 (7.1) | 8 |  |
| Enhancement degree |  |  |  | 0.180 |
| Hyperenhancement | 62 (78.5) | 26 (92.9) | 88 |  |
| Isoenhancement | 6 (7.6) | 0 (0) | 6 |  |
| Hypoenhance | 11 (13.9) | 2 (7.1) | 13 |  |
| Entirely wash-out time of lesions |  |  |  | 0.997 |
| ≤ 5mins | 31 (39.2) | 11 (39.3) | 42 |  |
| ＞ 5mins | 48 (60.8) | 17 (60.7) | 65 |  |
| Uptake pattern |  |  |  | 0.376 |
| Centripetal | 46 (58.2) | 13 (46.4) | 59 |  |
| Centrifugal | 2 (2.5) | 2 (7.1) | 4 |  |
| Diffuse | 31 (39.2) | 13 (46.4) | 44 |  |
| No enhance |  |  |  |  |
| Homogeneous pattern |  |  |  | 0.260 |
| Presence | 17 (21.5) | 9 (32.1) | 26 |  |
| Absence | 62 (78.5) | 19 (67.9) | 81 |  |
| Rim like enhancement |  |  |  | 0.440 |
| Presence | 71 (89.9) | 27 (96.4) | 98 |  |
| Absence | 8 (10.1) | 1 (3.6) | 9 |  |
| Claw shaped pattern |  |  |  | 0.143 |
| Presence | 47 (59.5) | 21 (75.0) | 68 |  |
| Absence | 32 (40.5) | 7 (25.0) | 39 |  |
| Perfusion defects |  |  |  | 0.961 |
| Presence | 25 (31.6) | 9 (32.1) | 34 |  |
| Absence | 54 (68.4) | 19 (67.9) | 73 |  |
| Lesion size compared with conventional ultrasound increased |  |  |  | 0.201 |
| Presence | 16 (20.3) | 9 (32.1) | 25 |  |
| Absence | 63 (79.7) | 19 (67.9) | 82 |  |
| Margin |  |  |  | 0.942 |
| Well defined | 22 (27.8) | 8 (28.6) | 30 |  |
| Poorly defined | 57 (72.2) | 20 (71.4) | 77 |  |
| Shape |  |  |  | 0.012 |
| Regular | 17 (21.5) | 13 (46.4) | 30 |  |
| Irregular | 62 (78.5) | 15 (53.6) | 77 |  |
| Nourishing vessels |  |  |  | 0.957 |
| Presence | 23 (29.1) | 8 (28.6) | 31 |  |
| Absence | 56 (70.9) | 20 (71.4) | 76 |  |
| **TIC parameters** | | | | |
| MeanLin | 201540.23±771665.67 | 14817.56±86711.91 | - | 0.212 |
| PE | 1015357.51±3901118.95 | 80264.78±526095.80 | - | 0.217 |
| RT (s) | 7.29±4.81 | 7.21±4.68 | - | 0.938 |
| TTP (s) | 10.95±6.95 | 10.17±6.79 | - | 0.607 |
| mTT (s) | 64.74±47.22 | 76.99±61.79 | - | 0.342 |
| FT (s) | 18.62±20.56 | 18.45±14.45 | - | 0.962 |
| WiAUC | 2712702.25±9631043.28 | 178213.17±1007258.88 | - | 0.176 |
| WiR | 360775.40±1448265.47 | 36110.05±261208.07 | - | 0.248 |
| WiPI | 645141.34±2479975.87 | 51382.58±337775.44 | - | 0.217 |
| WoAUC | 5209295.18±17984790.64 | 343196.03±1954077.40 | - | 0.164 |
| WoR | 145045.41±564313.87 | 13365.60±92959.60 | - | 0.230 |
| WiWoAUC | 7921997.14±27604937.08 | 521409.21±2960631.31 | - | 0.168 |

Note: CEUS: contrast enhanced ultrasound; MeanLin: the average contrast signal intensity; PE: peak enhancement; RT: rising time; TTP: time to peak; mTT: mean transit time; FT: fall time; WiAUC: Wash-in Area Under the Curve; WiR: Wash-in Rate; WiPI: Wash-in Perfusion Index; WoAUC: Wash-out Area Under the Curve; WoR: Wash-out Rate; WiWoAUC: WiAUC + WoAUC.

**Supplementary table 4.** Selected features of 107 patients in training and validation set.

|  | **Training set (n=79) (%)** | **Validation set (n=28) (%)** | **Total (n=107)** |
| --- | --- | --- | --- |
| **Location** |  |  |  |
| Upper outer quadrant | 25 (31.6) | 11 (39.3) | 36 |
| Upper inner quadrant | 11 (13.9) | 1 (3.6) | 12 |
| Lower outer quadrant | 6 (7.6) | 0 (0) | 6 |
| Lower inner quadrant | 21 (26.6) | 12 (42.9) | 33 |
| Others | 16 (20.3) | 4 (14.3) | 20 |
| **Shape** |  |  |  |
| Regular | 13 (16.5) | 12 (42.9) | 25 |
| Irregular | 66 (83.5) | 16 (57.1) | 82 |
| **Strip-shaped echoic** |  |  |  |
| Absence | 20 (25.3) | 9 (32.1) | 29 |
| Present | 59 (74.7) | 19 (67.9) | 78 |
| **Perfusion defects** |  |  |  |
| Presence | 25 (31.6) | 9 (32.1) | 34 |
| Absence | 54 (68.4) | 19 (67.9) | 73 |
| **mTT (s)** | 64.74±47.22 | 76.99±61.79 | - |
| **FT (s)** | 18.62±20.56 | 18.45±14.45 | - |

Note: CEUS: contrast enhanced ultrasound; TIC: time intensity curve; mTT: mean transit time; FT: fall time.

**Supplementary table 5.** Researches of ultrasound, MRI and mammography in HER2 expression of breast cancer.

| **Author** | **Journal** | **Year** | **Number of centers** | **Study design** | **Number of patients** | **Model features** | **HER2 expression** | **Results** |
| --- | --- | --- | --- | --- | --- | --- | --- | --- |
| Bene et al [1] | Cancers | 2022 | 1 | prospective | 72 | Hybrid sonovue -CEUS radiomic features | HER2 3+ | AUC 0.836 (0.73–0.913), 76.92% sensitivity, 84.75% specificity |
| Cui et al [2] | Journal of translational medicine | 2023 | 2 | retrospective | 489 | B-mode ultrasound radiomic features | HER2 3+ | AUC 0.844 (0.762-0.92 7) |
| Ferre et al [3] | Breast disease | 2023 | 1 | retrospective | 88 | B-mode ultrasound radiomic features | HER2 3+ | AUC 0.778, 71.4% sensitivity , 71.6% specificity |
| [Zhou](https://webvpn.shsmu.edu.cn/https/77726476706e69737468656265737421e0e243912234265e7d0a80e296592e7bb7d62ae2c192eb/?term=Zhou+J&cauthor_id=34856951) et al [4] | BMC Med Imaging | 2021 | 1 | prospective | 189 | B-mode ultrasound BI-RADS features | HER2 3+ | AUC 0.725, 64.71% sensitivity, 72.89% specificity |
| Bitencourt et al [5] | EBioMedicine | 2020 | 1 | retrospective | 311 | Clinical and MRI radiomic features | HER2 IHC 3+ vs IHC 2+ or 1+ to 2+ with HER2 gene amplification by FISH | AUC 0.974, 99.3% sensitivity, 81.3% specificity |
| This study | / | / | 17 | prospective | 140 | B-mode, sonazoid-CEUS and TIC features | HER2 3+ and 2+, 1+ | AUC 0.869 (0.715 - 0.958) and 0.747 (0.548 - 0.891), respectively |

**References**

1. Bene, I., et al., Radiomic Signatures Derived from Hybrid Contrast-Enhanced Ultrasound Images (CEUS) for the Assessment of Histological Characteristics of Breast Cancer: A Pilot Study. Cancers (Basel), 2022. 14(16).

2. Cui, H., et al., Radiogenomic analysis of prediction HER2 status in breast cancer by linking ultrasound radiomic feature module with biological functions. J Transl Med, 2023. 21(1): p. 44.

3. Ferre, R., et al., Machine learning analysis of breast ultrasound to classify triple negative and HER2+ breast cancer subtypes. Breast Dis, 2023. 42(1): p. 59-66.

4. Zhou, J., et al., Application of preoperative ultrasound features combined with clinical factors in predicting HER2-positive subtype (non-luminal) breast cancer. BMC Med Imaging, 2021. 21(1): p. 184.

5. Bitencourt, A.G.V., et al., MRI-based machine learning radiomics can predict HER2 expression level and pathologic response after neoadjuvant therapy in HER2 overexpressing breast cancer. EBioMedicine, 2020. 61: p. 103042.


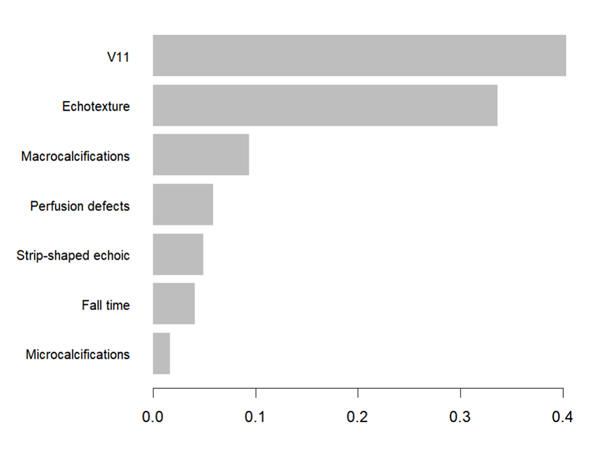


**Supplementary figure 1.** Selected variables in the combination of XGBoosting and Logistic regression model predicting HER2 3+ expression in descending order of contribution.
